# Supplementary material for: What matters to women in the postnatal period: A meta-synthesis of qualitative studies
Source: PLoS One. 2020 Apr 22;15(4):e0231415. doi: 10.1371/journal.pone.0231415 (PMC7176084; doi:10.1371/journal.pone.0231415)
Supplement: S1 Table — (DOCX) [file pone.0231415.s001.docx]

**Adapted PRISMA for reporting systematic reviews of qualitative and quantitative evidence**

| **Section/topic** | **Description** | |
| --- | --- | --- |
| **TITLE** | | |
| Title |  Propose a short take-home title. The title should explicitly  state that the review included different type of evidence. | See page 1 – the title reflects the focus of the study |
| **ABSTRACT** | | |
| Structured summary |  Provide a structured summary including, as applicable: background; objectives; data sources; study eligibility criteria, participants, and interventions; study appraisal and synthesis methods; results; limitations; conclusions and implications of key findings; systematic review registration number. | See Manuscript. |
| **INTRODUCTION AND OBJECTIVES** | | |
| Rationale |  Describe the rationale for the review (e.g., a health problem) in the context of what is already known (e.g., an existing literature review paper or a reference book chapter). | See pages 4 & 5 under Introduction |
| Objectives | Formulate questions and/or objectives (qualitative,  quantitative or both) being addressed by your review. | See page 5 under Introduction |
| **METHODS** | | |
| Protocol and registration |  Indicate if a review protocol exists, if and where it can be accessed (e.g., Web address), and, if available, provide registration information including registration number. | The study contributes to the World Health Organization’s (WHO) revised Postnatal Guidelines scheduled for publication in 2020 |
| Justification |  Justify the use of a review of qualitative and quantitative  evidence. | This review is only qualitative and was used to inform the above guidelines |
| Eligibility criteria |  Specify the inclusion and exclusion criteria and the rationale  for supporting these criteria. | See pages 6 & 7 |
| Information sources |  Describe all information sources (e.g., databases with dates of coverage, contact with study authors to identify additional studies) in the search and date last searched. | See pages 7 & 8 |
| Search | - Present full electronic search strategy for at least one database (e.g., in an appendix), including any limits used, such that it could be repeated. - Describe the process for removing duplicates. | See page 7 (Box 1)  See page 8 |
| Study selection |  Describe the process for selecting studies (e.g., screening based on titles and abstracts, and eligibility based on full-text, number of reviewers, software used). | See page 8 and PRISMA Flowchart (Fig 1) for details |
| Data collection process | - Describe the method of data extraction from included studies (e.g., number of reviewers involved, piloted forms, etc.). - List the data extracted. | See page 9 |
| Appraisal | - Describe the process for appraising included studies (e.g., tools used, number of reviewers involved), and specifically for assessing the methodological quality or risk of bias of included qualitative, quantitative, and mixed methods studies. - Specify how results of this appraisal are used in the synthesis. For example, for descriptive purpose (include all studies with description of their methodological quality or risk of bias) or for analytical purpose (contrast synthesis of ‘lower quality’ studies vs. ‘higher quality’ studies using sensitivity analysis). | See page 8 and Table 1  See page 9 for details of how the GRADE-CERQual tool was used to assess confidence in the findings. |
| Synthesis | - Describe the synthesis design used. Describe and justify the synthesis method(s) used (e.g., quantitative content analysis, meta-analysis, thematic synthesis, etc.). | See page 9. We used meta-ethnography to synthesize the findings. This is the most frequently used approach and is suitable for the studies identified |
| Additional analysis |  Describe methods of additional analyses (e.g., sensitivity or  subgroup analyses), if done. | No further analyses were conducted |
| **RESULTS** | | |
| Study selection |  Give numbers of studies screened, assessed for eligibility, and  included in the review, with reasons for exclusions at each | See PRISMA Flowchart (Fig 2) for details |

|  |  | stage. | |  |
| --- | --- | --- | --- | --- |
|  |  | Summarize in a flow diagram (see Appendices). | |  |
|  |  |  | |  |
| Study characteristics |  | For each study, present characteristics for which data were extracted (e.g., tables of characteristics of included studies – see Appendices) and provide the citations. | | See Table 1 for details of study characteristics |
|  |  | Specify common information across all included studies. | | See Table 2 |
|  |  | Describe the studies including their heterogeneity (variability associated with differences between studies). | | See Table 1 and pages 20 & 21 |
| Result of appraisal |  | Present data on the methodological quality or risk of bias of included studies based on the appraisal done. | | See quality appraisal and CERQual grading on Table 1 |
| Results of synthesis |  | Present results of synthesis. | | See Table 2 |
|  |  | If qualitative synthesis: In the text, briefly summarize the main themes or categories and refer to the appendix. | | See pages 20-25 for details |
|  |  | Appendix (table, figure, or matrix): For each study, present the themes or categories identified | | See Table 2 |
|  |  |  | |  |
| Additional analysis |  | Give results of additional analyses, if done (e.g., sensitivity or subgroup analyses). | | N/A |
| **DISCUSSION** | |  | | |
| Summary of evidence |  | Provide an overall summary of results (take-home messages) from the qualitative and/or quantitative synthesis. | | See page 22 for line of argument synthesis |
|  |  | State the main results for each main theme or category, and/or key process/outcome variable. | | See Table 2 |
|  |  | Consider their relevance and importance for knowledge users (e.g., health care providers, managers, and decision/policy makers). | | See pages 21-25 |
|  |  | Take into account the methodological quality across studies (when applicable). | | See Quality appraisal and CERQual gradings in Table 1 |
|  |  | Describe insight gained from the integration of qualitative and quantitative evidence. | | N/A |
| Contribution |  | Describe the contribution of the review (compared to what is already known) with respect to Review methods, Scientific knowledge, Practice, program planning and evaluation, policy making, or anything else | | See pages 32-36 |
| Limitations |  | Specify any element that may affect the cumulative evidence. | | N/A |
|  |  | Discuss limitations at the study and process/outcome levels (e.g., lack of rich data for qualitative synthesis, methodological quality/risk of bias, and their potential consequences on the results). | | See pages 35 & 36 |
|  |  | Discuss limitations at the review level (e.g., dependent reviewers, incomplete retrieval of relevant studies - selective publication of reports regarding studies with positive results), and limited reporting (selective reporting of information about included studies)), and their potential consequences on the results. | | See pages 35 & 36 |
| Conclusions |  | Provide a general interpretation of the results in the context of other evidence, including implications for knowledge users (e.g., major recommendation). | | See pages 32-36 |
|  |  | State implications for future research. | | See pages 35 & 36 |
| **ACKNOWLEDGEMENTS** | |  | | |
| Acknowledgements |  | Describe sources of funding and other support (e.g., supply of data) and the role of funders in the review. | | See page 3 for funding information and 36 for further acknowledgements |
|  |  | Acknowledge any information about potential conflict of interest. | | Included in journal submission. No conflict of interest declared |
| **REFERENCES** | | | | |
| References |  List all the references cited in the text. | | | See pages 37-45 |
| **APPENDICES (tables, figures, boxes, …)** | | | | |
| Flow diagram | 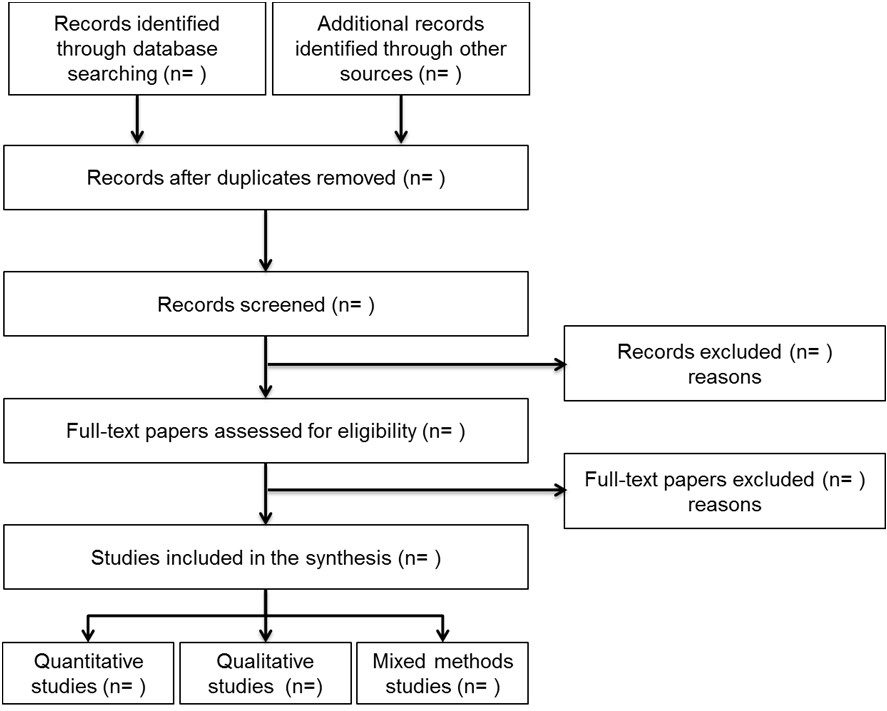 | | | |
| Table of characteristics of included studies | • Summarize key characteristics of the included studies in a table such as:  - Source (first author, year),  - Study design,  - Number of participants and participants (e.g., age, sex,  other),  - Setting (e.g., types and number of organizations),  - Intervention/exposure (if applicable), or relevant  considerations for non-intervention studies, - Outcomes (if applicable), or relevant considerations for  non-intervention studies,  - Quality rating (if applicable),  - Further comments on key characteristics of the study  (according to the review question),  Different tables may be needed: For example, tables for quantitative studies, for qualitative studies, and for mixed methods studies. | | See Table 1 for characteristics of included studies | |
| Tables and figures on results of the synthesis |  Provide illustrations for results of the synthesis such as:   - Quantitative synthesis: statistical summary, descriptive table, forest plots, etc. - Qualitative synthesis: list of categories, list of themes and subthemes, concept maps, framework, etc. | | N/A  See Table 2 | |

*Adapted from:*  Moher D, Liberati A, Tetzlaff J, Altman DG, The PRISMA Group (2009). Preferred Reporting Items for Systematic Reviews and Meta-Analyses: The PRISMA Statement. PLoS Med 6(6): e1000097. doi:10.1371/journal.pmed1000
